# Predator-induced transgenerational plasticity of parental care behaviour in male three-spined stickleback fish across two generations

**DOI:** 10.1098/rspb.2023.2582

**Published:** 2024-01-10

**Authors:** Jennifer K. Hellmann, Jason Keagy, Erika R. Carlson, Shayne Kempfer, Alison M. Bell

**Affiliations:** ^1^ Department of Evolution, Ecology and Behavior, School of Integrative Biology, University of Illinois Urbana-Champaign, Urbana, IL 61801, USA; ^2^ Carl R. Woese Institute for Genomic Biology, University of Illinois Urbana-Champaign, Urbana, IL 61801, USA; ^3^ Program in Ecology, Evolution and Conservation, University of Illinois Urbana-Champaign, Urbana, IL 61801, USA; ^4^ Department of Evolution, Ecology, and Organismal Biology, The Ohio State University, Columbus, OH 43210, USA; ^5^ Department of Ecosystem Science and Management, Pennsylvania State University, University Park, PA 16802, USA

**Keywords:** paternal effect, paternal care, *Gasterosteus aculeatus*, transgenerational plasticity, epigenetics, plasticity

## Abstract

Parental care is a critical determinant of offspring fitness, and parents adjust their care in response to ecological challenges, including predation risk. The experiences of both mothers and fathers can influence phenotypes of future generations (transgenerational plasticity). If it is adaptive for parents to alter parental care in response to predation risk, then we expect F_1_ and F_2_ offspring who receive transgenerational cues of predation risk to shift their parental care behaviour if these ancestral cues reliably predict a similarly risky environment as their F_0_ parents. Here, we used three-spined sticklebacks (*Gasterosteus aculeatus*) to understand how paternal exposure to predation risk prior to mating alters reproductive traits and parental care behaviour in unexposed F_1_ sons and F_2_ grandsons. Sons of predator-exposed fathers took more attempts to mate than sons of control fathers. F_1_ sons and F_2_ grandsons with two (maternal and paternal) predator-exposed grandfathers shifted their paternal care (fanning) behaviour in strikingly similar ways: they fanned less initially, but fanned more near egg hatching. This shift in fanning behaviour matches shifts observed in response to direct exposure to predation risk, suggesting a highly conserved response to pre-fertilization predator exposure that persists from the F_0_ to the F_1_ and F_2_ generations.

## 1. Introduction

Across a wide range of taxonomic groups, organisms exhibit a range of behaviours that are associated with improving the growth and survival of their offspring [1]. For many organisms, parental care is a critical determinant of offspring fitness, altering survival, growth, cognition and anti-predator responses [2–5]. These fitness benefits arise partly because parents can adjust their care in response to ecological challenges (e.g. temperature [6–8], predation risk [9,10]), which may buffer offspring by mitigating harsh early life conditions or by increasing offspring resilience to environmental stressors. This plasticity in parental care may therefore allow organisms to occupy harsher environments than they could have in the absence of this care [7].

While individuals adjust their care based on ecological conditions encountered in their own lifetime, we know less about how individuals may alter their care based on the ecological conditions encountered by their parents or even grandparents. There is growing appreciation that both maternal and paternal environments can influence the phenotypes of future generations (transgenerational plasticity (TGP) or environmental parental effects). TGP is widespread across different taxonomic groups [11,12] and can be adaptive when it allows parents to programme their offspring for the environment they are likely to experience [13,14]. TGP can occur in response to a variety of environmental cues, including predation risk [15–17], pathogens [18,19], environmental toxins [20,21] and warming temperatures [22]. These parental environments can alter offspring phenotypes via multiple mechanisms starting from the time of fertilization (e.g. hormones or nutrients in eggs or seeds, small RNAs in gametes) into the post-emergence period (e.g. parental care) [13,23,24]. These phenotypic alterations can persist for multiple generations, even if the subsequent generations are reared in the absence of cues that triggered a response in the F_0_ generation [13]. This may be because environmental influences can alter epigenetic marks (e.g. small non-coding RNAs and methylation in gametes) that might be inherited across multiple generations via effects on the germ line [13,24–26], although these mechanisms are still not well understood.

Here, we used three-spined sticklebacks (*Gasterosteus aculeatus*) to understand how paternal experience with predation risk alters reproductive traits and parental care behaviour in F_1_ sons and F_2_ grandsons. For paternal effects, only phenotypic changes in the F_2_ generation and beyond are considered truly transgenerational, as any environment experienced by F_0_ males simultaneously exposes both the father (F_0_) and his germline (future F_1_; [27]). Three-spined sticklebacks have *male-only* parental care, paternal care is an ecologically important behaviour with fitness consequences and the quality of paternal care given can have important effects on offspring traits [28,29]. Fanning embryos is a critical care behaviour, but is high risk because it is conspicuous, potentially drawing predator attention and making anti-predator vigilance more difficult (e.g. the head is pointed into the nest, reducing the area that can be visually scanned). Previous studies suggest that fanning behaviour is responsive to predation risk: fathers exposed to predation risk while providing care (3 days post-fertilization) decrease the amount of care provided for several days afterward [10]. Similarly, pre-fertilization exposure to predation risk causes fathers to take longer to mate and to shift their paternal care behaviour by reducing fanning early in egg development (directly after predation exposure) but then increasing fanning right before egg hatching [30]; this may be an adaptive mechanism to avoid giving care until it is clear that the clutch is viable (embryos develop notable eye spots around 3 days post-fertilization).

We exposed F_0_ male sticklebacks to simulated predation risk prior to fertilization. We then used *in vitro* fertilization and artificial incubation to generate F_1_ offspring of control and predator-exposed fathers and F_2_ grandsons with either two control grandfathers, a predator-exposed maternal grandfather, a predator-exposed paternal grandfather or two predator-exposed grandfathers. We manipulated exposure across maternal and paternal lineages to understand if epigenetic changes to paternal reproductive traits need to be mediated via paternal lineages (via F_1_ father to F_2_ son) or can also be mediated via maternal lineages (via F_1_ mothers to F_2_ sons). For both the F_1_ and F_2_ generation, we reared males to adulthood in the absence of predation risk and then measured them for reproductive traits, including throat coloration, latency to nest/mate and parental care behaviour.

Using artificial incubation ensures that the cue is inherited via sperm/seminal fluid (sperm-mediated) and controls for the potential effects of the early life environment and the possibility that the quality of parental care received influences subsequent parental behaviour [31–34]. Recent studies have shown that intergenerational effects of male exposure to predation risk *prior* to fertilization can be mediated via sperm alone [30]; these sperm-mediated paternal effects alter a variety of traits in the F_1_ and F_2_ generations, including anti-predator behaviour, survival, size and stress responsiveness [30,35–37]. If sperm-mediated paternal cues of predation risk alter reproductive traits, we expect F_1_ and F_2_ descendants of predator-exposed F_0_ males to show the same behavioural shifts as directly exposed F_0_ males: relative to control males, F_1_ and F_2_ progeny of predator-exposed F_0_ males will take longer to mate and shift their paternal care behaviour by reducing fanning early in egg development and increasing fanning before egg hatching [30]. If this can only be mediated from fathers to sons, then we would expect that these effects would persist to the F_2_ generation when F_2_ males have a predator-exposed paternal grandfather, but not a predator-exposed maternal grandfather. Finally, if information becomes less reliable across generations, we might expect that the effects attenuate over generations (i.e. stronger effects in the F_1_ compared to the F_2_ generations) or that we see a response in F_2_ males only if *both* grandfathers were exposed.

## 2. Methods

### (a) Housing conditions

In both summer 2016 and 2017, sexually mature three-spined sticklebacks were collected from Putah Creek, a freshwater stream in northern California, and shipped to the University of Illinois at Urbana-Champaign. This population has piscivorous predators, including the prickly sculpin (*Cottus asper*). F_0_ fish were maintained on a summer photoperiod schedule (16 L : 8 D) at 21° ± 1°C and fed *ad libitum* daily with a mix of frozen bloodworms (*Chironomus* spp.), brine shrimp (*Artemia* spp.), mysis shrimp (with *Spirulina* algae) and cyclopod + (primarily *Oithona similis*). All generations in this experiment were housed on recirculating temperature-controlled flow-through racks in the same room, with particulate, biological and UV filters; this allowed for conditions to be standardized among tanks during nesting, egg incubation and fry rearing. Note that as F_0_ individuals used for the F_1_ and F_2_ generations were collected from the wild in different years, it is highly likely that the F_1_ and F_2_ generations are unrelated to each other and represent distinct genetic lineages (i.e. we had a large pool of genetic diversity).

### (b) Generating the F_1_ generation

During August to November 2016 and October to November 2017, we bred wild-caught (F_0_) males and females to generate F_1_ fish. F_0_ males were transferred to individual 26.5 l ‘nesting' tanks (36 L × 33 W × 24 H cm) with gravel, two plastic plants, a plastic box filled with sand, a clay pot and algae to encourage nest building. The sides and back of the tank were covered with opaque barriers to block other tanks from view. Once F_0_ males had successfully built a nest, they were either exposed to a clay model sculpin (21 cm long) six times over 11 days (30 s each) or left undisturbed during an equivalent time frame. This predator exposure regimen was designed to mimic conditions that males experience when they move into shallow habitats to nest. We elected to expose males to a relatively short stressor in order to minimize the potential for males to habituate to the model predator [38]. Stickleback males produce sperm in the beginning of the breeding season [39]; thus, paternal effects mediated via sperm in this experiment are likely due to epigenetic modifications to already mature sperm or seminal fluid. We found no differences in fertilization rates among treatment groups for either the F_1_ [36] or F_2_ [37] generation.

The day after the last exposure, we removed the male, extracted his testes, and used his sperm to fertilize eggs of a wild-caught, unexposed female. We placed fertilized eggs in a cup with a mesh bottom above a bubbler. Once hatched, offspring were fed newly hatched brine shrimp naupilii for two months before transitioning to adult food (as above) with an intermediate stage using small fragments of adult food. Offspring were switched to a winter light schedule (8 L : 16 D) within two months of hatching. F_1_ fish generated in 2016 were used to generate an F_2_ generation in 2017 (which were assayed for parental care in 2018), whereas F_1_s generated in 2017 were assayed for parental care in 2018 (see below).

### (c) Generating the F_2_ generation

**Figure 1.**
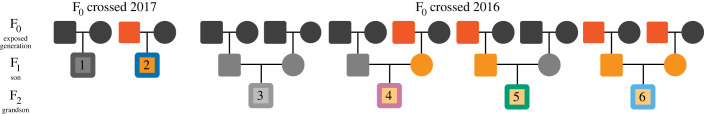
Overview. The pedigrees track the pattern of predation exposure in parents or grandparents of all treatment groups (1–6). Squares indicate males and circles indicate females. There were two treatment groups for sons: (1) males whose fathers had not been exposed to predators or (2) males whose fathers had been exposed to predators prior to fertilization. There were four treatment groups for grandsons: (3) neither grandfather exposed, (4) maternal grandfather exposed, (5) paternal grandfather exposed, and (6) both grandfathers exposed. Shading represents generational time since the initial experiment involving predator exposure, i.e. most dark for experienced by the individual and most light for experienced by the grandfather(s). Black signifies control and orange signifies predation exposure. The outline of the sons and grandsons corresponds to the colours used in the data figures.

From August to October 2017, we bred F_1_ males and females to generate the F_2_ generation. We isolated adult F_1_ sons of control and predator-exposed fathers in 26.5 l ‘nesting’ tanks set up as described above to confirm reproductive readiness. Upon completing his nest, we euthanized the male to obtain his sperm. We maintained F_1_ daughters of control and predator-exposed fathers in their home tanks (to mimic shoaling in the wild); once females were gravid, we gently squeezed their abdomen to obtain eggs. Husbandry and care methods were parallel to the F_1_ generation (for more details, see Hellmann *et al*. [37]). We generated four different F_2_ treatment groups (figure 1): F_2_s with (i) two control grandfathers (offspring of F_1_ daughters and sons of control males), (ii) a predator-exposed maternal grandfather (offspring of F_1_ daughters of predator-exposed males and F_1_ sons of control males), (iii) a predator-exposed paternal grandfather (offspring of F_1_ daughters of control males and F_1_ sons of predator-exposed males) or (iv) two predator-exposed grandfathers (offspring of F_1_ daughters and sons of predator-exposed males). Both F_1_ and F_2_ generations were reared in the absence of predation risk (i.e. the only generation exposed to a model predator was the F_0_ generation) and were left undisturbed prior to mating or assessing parental care (i.e. they were not removed from their nesting tank for any measurements or assays).

### (d) Parental care observations

In May to November 2018, we observed parenting behaviour of F_1_ (generated from males wild caught in 2017) and F_2_ males (descended from males wild caught in 2016). For all treatments (figure 1), males were transferred to individual 26.5 l nesting tanks set up as described above (mean = median = 17.5 fish, mode = 17 fish, range 14–20 fish for each treatment group, table 1). Just prior to adding a male to his tank, we evaluated the extent of his red or orange throat coloration (a sexually selected trait important for male competition and female choice [40,41]) using a 0–5 scale with 0.5 increments [42,43]. Once a male successfully built a nest, we introduced a gravid, wild-caught female into the tank; she remained in the tank until she successfully laid eggs in the male's nest or up to 2 h. We ran up to two courtship trials per day (one morning and one afternoon), depending on the availability of gravid females.

**Table 1.** Number of fish that did or did not reach each stage of reproduction.

	F_1_ control father	F_1_ predator-exposed father	F_2_ control GFs	F_2_ predator-exposed maternal GF	F_2_ predator-exposed paternal GF	F_2_ two predator-exposed GFs
total	14	17	18	17	19	20
built nests	11	14	16	16	14	18
mated	9	11	11	11	12	11
raised offspring	6	5	6	5	6	5

Once a male mated, the front of his tank was covered with an opaque barrier to minimize disturbance. A tilted mirror was placed on the top of the tank to enable behavioural observations without having to remove the barrier. We observed paternal care behaviours for 5 min d^−1^, beginning the day of fertilization (within 2 h of fertilization) until 8 days post-fertilization (nine observations total; electronic supplementary material, figure S1); we conducted observations around the same time every day (if the male mated in the morning, observations were conducted between 09.00 and 13.00; if the male mated in the afternoon, observations were conducted between 13.00 and 17.00). Although the use of the mirror minimized potential disturbance of the male by the observer, observers also included a 5 min acclimation period prior to beginning their observation of the male.

For each observation, we recorded the amount of time the male fanned his nest. Fanning, in which the father aerates the eggs and recently hatched fry (eggs hatch about 6 days post-fertilization), is the most common parenting behaviour shown by sticklebacks and varies with predation risk [10,44]. Fanning behaviour is highly repeatable and 5 min samples are adequate for describing daily variation between males in this behaviour [45]. For example, in Bell *et al*. [45], we recorded 1 h on the day of peak fanning and then subsampled every 5 min and found high consistency among 5 min subperiods (the fish fanned 41.55% of that hour and the average deviation from this for a given 5 min sample was only 0.19% with a standard deviation of 4.61%).

We gently removed males from the tank after the last observation. We weighed and measured the standard length (tip of nose to end of caudal peduncle) of males, and then once again evaluated the extent of their throat coloration. Note that these methods for observing parental behaviour are identical to those used in Hellmann *et al*. [30], where we observed parental care behaviour of *directly* exposed F_0_ males during the same time period (September to November 2018). Sample sizes for F_1_ and F_2_ fish at each stage of this process from transfer into the nesting tank to being observed for parental care can be found in table 1.

### (e) Statistical analysis

All statistical analyses were performed using R version 4.0.1 [46]. We used two-sample *t*-tests and one-way ANOVAs to test for differences between F_1_ and F_2_ treatments, respectively, in throat colour (before and after parental care), (log-transformed) days to build a nest, (log-transformed) number of mating attempts, standard length (after parental care) and mass (after parental care). Assumptions of *t*-tests and one-way ANOVAs (e.g. equality of variance) were first tested; variance was not equal for F_1_ fish mass and so a Welch two-sample *t*-test was used for that analysis. We used *G*-tests (*GTest* function in *DescTools* package [47]) to test for differences between F_1_ and F_2_ treatments in likelihood to progress from one stage of reproduction to the next (build a nest, mate and raise offspring to independence).

For those males that raised offspring to independence (i.e. had visible fry in the tank, range five to six males per treatment group), we analysed fanning behaviour with linear mixed effects models (*lmer* function in the *lme4* package, [48] with Satterthwaite approximation of degrees of freedom for *t*-tests of model coefficients (*anova* function in the *lmerTest* package, [49])). Because fanning increases until around day 5 post-fertilization (roughly around the time of hatching) and then decreases, we included both a linear effect of day and a quadratic effect of day as fixed effects. We also included a fixed effect of treatment, and interactions between treatment with both the linear and quadratic effect of day (0–8 days with 1 day increments) to understand if the shape of the fanning curve varied with treatment. To account for repeated measures, we included paternal ID as a random effect. We also included observer ID as a separate random effect to account for any potential differences between scoring fanning behaviour. This random effect never explained any variance (tables 2 and 3), probably because fanning is a very obvious behaviour with very distinct beginning and ending—the fins of the father are either moving or not. Residuals of linear mixed effects models were normally distributed.

**Table 2.** Results of a quadratic generalized linear mixed model of the amount of time F_1_-generation fathers fanned their nest over time. The impact of treatment (two levels, with control fish as the reference level) on the fanning curve was modelled, including the impact on intercept and the linear and quadratic time components. The male identity and the observer recording behaviour were included as random effects. Italicized if 0.1 < *p* < 0.05. Bold if *p* < 0.05. *N* = 99 observations, 11 males, seven observers.

fixed effects	estimate	s.e.	d.f.	*t-*value	*p*-value
(intercept)	5.36	12.73	58.60	0.42	0.68
*F*_1_ *treatment*	*−35*.*32*	*18*.*89*	*58*.*60*	*−1*.*87*	*0*.*07*
*(exposed father)*					
**day**	**41**.**73**	**6**.**69**	**84**.**00**	**6**.**23**	**<0**.**001**
**day^2^**	**−5**.**15**	**0**.**81**	**84**.**00**	**−6**.**39**	**<0**.**001**
F_1_ **treatment × day**	**25**.**71**	**9**.**93**	**84**.**00**	**2**.**59**	**0**.**011**
**(exposed father)**					
F_1_ **treatment × day^2^**	**−2**.**45**	**1**.**19**	**84**.**00**	**−2**.**05**	**0**.**043**
**(exposed father)**					
random effects	variance				
male ID	181.4				
observer	0.0				
residual	1198.0

**Table 3.** Results of a quadratic generalized linear mixed model of the amount of time F_2_-generation fathers fanned their nest over time. The impact of treatment (four levels, with control fish as the reference level) on the fanning curve was modelled, including the impact on intercept and the linear and quadratic time components. The male identity and the observer recording behaviour were included as random effects. Italicized if 0.1 < *p* < 0.05. Bold if *p* < 0.05. *N* = 197 observations, 22 males, eight observers.

fixed effects	estimate	s.e.	d.f.	*t*-value	*p*-value
(intercept)	6.18	16.87	87.08	0.37	0.71
F_2_ treatment	9.28	25.05	87.32	0.37	0.71
(maternal GF-exposed)					
F_2_ treatment	−8.05	23.86	87.08	−0.34	0.74
(paternal GF-exposed)					
F_2_ treatment	−28.79	25.02	87.08	−1.15	0.25
(both GF-exposed)					
**day**	**40**.**56**	**8**.**27**	**167**.**05**	**4**.**90**	**<0**.**001**
**day^2^**	**−4**.**64**	**0**.**99**	**167**.**05**	**−4**.**67**	**<0**.**001**
F_2_ treatment × day	4.88	12.36	167.19	0.40	0.69
(maternal GF-exposed)					
F_2_ treatment × day	4.22	11.70	167.05	0.36	0.72
(paternal GF-exposed)					
F_2_ **treatment** × **day**	**26**.**21**	**12**.**27**	**167**.**05**	**2**.**14**	**0**.**034**
**(both GF-exposed)**					
F_2_ treatment × day^2^	−1.08	1.49	167.17	−0.72	0.47
(maternal GF-exposed)					
F_2_ treatment × day^2^	−0.26	1.41	167.05	−0.18	0.86
(paternal GF-exposed)					
F_2_ **treatment** × **day^2^**	**−2**.**93**	**1**.**48**	**167**.**05**	**−1**.**99**	**0**.**049**
**(both GF-exposed)**					
random effects	variance				
male ID	499.1				
observer	0.0				
residual	1828.9				

## 3. Results

### (a) Throat coloration of F_1_ or F_2_ fish was not altered by F_0_ predator exposure

We did not detect significant differences in the extent of throat coloration between F_1_ sons of control versus predator-exposed fathers, either before parenting (two-sample *t*-test: *t*_29_ = −0.14, *p* = 0.89) or after parenting (two-sample *t*-test: *t*_9_ = 0.18, *p* = 0.86). We also did not detect significant differences between different F_2_ treatment groups of fish in the extent of throat coloration, either before parenting (one-way ANOVA: *F*_3,70_ = 0.93, *p* = 0.43) or after parenting (one-way ANOVA: *F*_3,18_ = 0.25, *p* = 0.85).

### (b) F_1_ or F_2_ fish did not differ in likelihood of nesting, mating, or successfully raising offspring based on F_0_ predator exposure

We did not detect significant differences between fish based on their father's or grandfather's predator-exposure in likelihood to build a nest (F_1_: *G* = 0.07, d.f. = 1, *p* = 0.79; F_2_: *G* = 3.54, d.f. = 3, *p* = 0.32), mate (F_1_: *G* = 0.04, d.f. = 1, *p* = 0.84; F_2_: *G* = 2.56, d.f. = 3, *p* = 0.46), or successfully raise offspring to independence (F_1_: *G* = 0.91, d.f. = 1, *p* = 0.34; F_2_: *G* = 0.25, d.f. = 3, *p* = 0.97; table 1 for sample sizes). F_1_ fish who built nests took a similar number of days to finish construction regardless of whether their fathers were exposed to predation or not (two-sample *t*-test: *t*_23_ = 1.16, *p* = 0.26). We did not detect significant differences in nest construction time between the different F_2_ treatment groups of fish (one-way ANOVA: *F*_3,60_ = 0.93, *p* = 0.43).

### (c) F_0_ predation exposure resulted in F_1_ fish taking longer to mate

Of those fish that mated, F_1_ fish with predator-exposed fathers took more attempts to mate than those with control fathers (two-sample *t*-test: *t*_18_ = −2.21, *p* = 0.040). These differences did not persist to the F_2_ generation (one-way ANOVA: *F*_3,41_ = 0.17, *p* = 0.91).

### (d) F_0_ predation exposure did not alter the size of F_1_ and F_2_ parenting males

There were no differences in size of males who successfully parented, for either the F_1_ fish (standard length: two-sample *t*-test, *t*_9_ = 1.27, *p* = 0.24; mass: Welch two-sample *t*-test, *t*_7.70_ = 1.43, *p* = 0.19) or F_2_ fish (one-way ANOVA; standard length: *F*_3,18_ = 1.52, *p* = 0.24 or mass: *F*_3,18_ = 1.14, *p* = 0.36).

### (e) F_0_ predation exposure altered F_1_ and F_2_ parental care (fanning) behaviour

**Figure 2.**
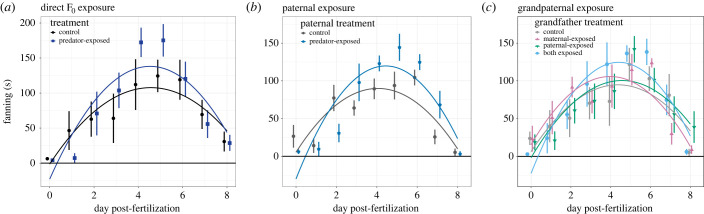
Transgenerational effects of predator exposure on fanning. Fish with predator-exposed fathers (*b*) start out with less fanning (indicated by the marginal treatment term and the significant and negative treatment × day^2^ term) but then have a steeper increase in fanning and end up with a peak that is higher (indicated by the significant and positive treatment × day term). This is a similar shift in care that is found in directly exposed F_0_ males (*a*; figure produced from data from Hellmann *et al*. [30]). F_2_ males (*c*) with two predator-exposed grandfathers (pale blue) had different fanning curves than those with two control grandfathers (grey); their fanning curves were very similar to that of fish with predator-exposed fathers and directly predator-exposed males. Fish with just one predator-exposed grandfather did not differ from those with two control grandfathers. Mean ± s.e. plotted for each day, curves are those predicted by the linear model, averaged for each treatment.

We found strong evidence that parental care (fanning) of F_1_ males differed based on whether their fathers were predator-exposed or not, and parental care of F_2_ males differed based on whether their grandfathers were predator-exposed or not; specifically, males who had *both* grandfathers exposed to predation behaved differently from the control F_2_ males. The shape of the fanning curves differed between these treatments; in other words, paternal predation exposure in the F_0_ generation affected how the fanning behaviour of F_1_ and F_2_ males was partitioned across the parenting days (tables 2 and 3, figure 2*b,c*). Specifically, the peak of fanning behaviour was later, but higher, for F_1_ males with predator-exposed fathers relative to control F_1_ males and for F_2_ males with two predator-exposed grandfathers relative to control F_2_ males (i.e. there was a positive coefficient for the corresponding F_1_/F_2_ treatment by day interaction, tables 2 and 3). In addition, F_1_ males with predator-exposed fathers and F_2_ males with two predator-exposed grandfathers fanned less in the beginning of the observation period (i.e. the shape of the curve was narrower/steeper in F_1_ and F_2_ males with predator-exposed F_0_ males compared to controls, with a negative coefficient for the F_1_ or F_2_ treatment by day^2^ interaction, tables 2 and 3). This shift in paternal care is similar to that observed when fathers are directly exposed to predation risk prior to fertilization [30] (figure 2*a*). This demonstrates that F_1_ males altered their paternal care behaviours in response to perceived predation risk *of their fathers*. Further, F_2_ males altered their paternal care behaviours in response to perceived predation risk *of their grandfathers*, but only if *both* grandfathers had been exposed to predators. Despite the shifts in fanning behaviour, we did not detect significant differences among treatments in the overall amount of fanning. For example, there was only a non-significant trend for F_1_ fish with predator-exposed fathers to fan less overall than F_1_ fish with control fathers (difference in intercept between treatments, *p* = 0.066; table 2 and figure 2*b*); the overall amount of fanning did not vary between control F_2_ males and any of the other treatments (non-significant differences in intercept, all *p* > 0.25; table 3 and figure 2*c*).

## 4. Discussion

Empirical studies and theory to date have primarily focused on maternal effects; however, there is growing evidence that the environment experienced by fathers can also affect the phenotypes of future generations [13,24]. Fathers have at least two mechanisms by which they can influence offspring non-genetically: epigenetic changes to sperm/seminal fluid (sperm-mediated) and behavioural changes to parental care (behaviourally mediated) [24]. Here, we explored how a brief paternal exposure to predation risk in the F_0_ generation induced changes in reproductive traits in his unexposed F_1_ and F_2_ male progeny. In our experiment, neither the F_1_ nor the F_2_ generation received parental care, allowing us to completely isolate how predator-induced transgenerational effects mediated via sperm altered care behaviours while controlling for early life experiences (e.g. the quality of care received).

We found that F_1_ sons of predator-exposed fathers took more trials to mate successfully compared to F_1_ sons of control fathers. However, these differences did not persist to the F_2_ generation. This is parallel to another study which found that males who were directly exposed to predation risk took more attempts to mate compared to control males [30]. Further, this is similar to another study which found that when females were exposed to predation risk, males courted those females less, implying they found them less attractive or that the females were less receptive to their courtship [50]. Given that we found no difference in coloration between sons of control or predator-exposed males, if this delay in mating was due to lower attractiveness of the males, it was likely linked to a behavioural or olfactory difference between males with control and predator-exposed fathers, or a difference in male motivation to mate based on paternal experiences.

We found larger and more consistent effects of F_0_ predator exposure when we examined the pattern of paternal care (fanning) behaviour in sons and grandsons. F_1_ and F_2_ males shift their fanning behaviour in strikingly similar ways: F_1_ males with predator-exposed fathers and F_2_ males with two predator-exposed grandfathers fanned less initially, but then rapidly increased their fanning with a higher peak amount of fanning when eggs were nearing the end of their development and about to hatch (e.g. a 38% and 31% increase on day 5 for F_1_ males with predator-exposed fathers and F_2_ males with two predator-exposed grandfathers compared to controls). Similar results were found in a recent study where males were directly exposed to predation risk prior to fertilization [30]. Thus, there is a remarkably consistent effect of pre-fertilization predator exposure that persists from the F_0_ to the F_1_ and F_2_ generations. Interestingly, although the effects are present in the F_2_ fish only if *both* grandfathers were exposed, we found no evidence that the effect attenuated between generations.

We originally suggested that this change in fanning in the directly exposed F_0_ males may reflect a period of time in which the father was recovering from pre-fertilization predation risk [30], consistent with Stein & Bell [10]. If this was the primary explanation for this behaviour, we would not expect the shift to persist in F_1_ and F_2_ males that were not directly exposed to predation risk. Instead, we suggest that this shifted fanning may reflect an adaptive behaviour in high-risk environments to minimize investment in clutches before it becomes clear that eggs have fertilized and are viable (eggs would likely fail to develop highly visible eyespots by day 3 post-fertilization if they were not viable). Fathers may then compensate for this initial reduced care by maximizing care during the days immediately prior to hatching (days 4–5 post fertilization) when fanning appears to be most critical. Interestingly, Foster *et al.* [51] show (in their fig. 3) that wild limnetic fish that were under predation risk from piscivores had a similar shift in fanning compared to benthic fish without piscivorous predation risk. Although the mechanism causing this population difference is not known (e.g. genetic, epigenetic, both) and their statistical analysis differed from ours, their results suggest our findings are generalizable even if we cannot make a direct comparison.

We observed a shift in fanning behaviour that persisted through the F_2_ generation when fathers had no opportunity to interact behaviourally with their offspring, suggesting this initial F_0_ cue was first inherited and then persisted through to the unexposed F_2_ generation via effects on the germline. Given that a sperm-mediated cue affected parental care behaviour, this suggests the potential for cross-mechanism effects, whereby cues passed via one non-genetic mechanism induce changes in a second non-genetic mechanism. This has important implications for predicting both the frequency and persistence of TGP in two ways. First, our results open the intriguing possibility that TGP could persist from generation to generation via a different mechanism than what initially induced the effect. This is supported by a previous finding by Gapp *et al*. [52] that behavioural symptoms persisted to their F_3_ generation even in the absence of the changes in sperm miRNAs that initially triggered the changes in the F_2_ generation. Further, this is supported by our data: because the cue was mediated to the F_2_ generation via F_1_ females (maternal grandfathers in the both grandfather treatment group) as well as F_1_ males, this suggests that the mechanism of transmission from the F_1_ to F_2_ generation could not have been via sperm alone. This cross-mechanism effect might be particularly likely to occur in species where offspring learn parental care from their parents; for example, sperm-mediated cues initially alter F_1_ parental care behaviour and F_2_ offspring learn to provide care similar to the care that they receive [31–34]. Second, the fact that a cue can be passed via one mechanism and then (either directly or indirectly) alter other TGP mechanisms could explain why transgenerational effects can accumulate across generations (i.e. the phenotype of the F_2_ or F_3_ generation exceeds the value of the phenotype induced in the F_1_ generation) [13]: something that is initially passed to the F_1_ generation via only one mechanism (sperm) could persist to the F_2_ generation via multiple mechanisms (sperm and paternal care). We did not explicitly test either of these implications, but it is a clear priority for future research. Collectively, these results strongly suggest that predicting both the frequency and persistence of TGP requires a better understanding of how one TGP mechanism can induce changes in other TGP mechanisms.

We initially outlined several patterns we might expect for TGP to manifest in F_1_ and F_2_ progeny of predator-exposed F_0_ males. We proposed we might see a shift in parental care to be inherited only from fathers to sons as, for example, with a Y-linked trait. However, we found that F_2_ males only displayed altered paternal care behaviour when the effect was mediated via both grandfathers, suggesting that in this case it also needed to be inherited via daughters of predator-exposed fathers to elicit the phenotype in the F_2_ generation. This might suggest that the cue of predation risk in the F_0_ generation is not altering paternal care directly (as females do not care), but instead altering traits that affect paternal care behaviours, such as anxiety or body condition [34]. In another study, we did not observe any differences in growth or behaviour in F_2_s with two predator-exposed grandfathers, although differences could arise in traits that we did not measure [37]. Alternatively, the cue could alter reproduction directly, with females as silent carriers of the epigenetic cue [37,53–55] or displaying altered reproductive traits that we did not measure here. Indeed, there is some evidence that parental predation exposure can alter mating preferences in daughters; for example, Lehto & Tinghitella [56] found that either stickleback mothers or fathers exposed to predation risk had daughters who preferred less conspicuous males (duller with less courtship). Future studies should investigate if these changes in female reproductive traits persist through to the F_2_ generation. Further, it would be fascinating to document how changes in F_1_ or F_2_ female preferences interact with changes in F_1_ or F_2_ male reproductive traits and parental care; if, for example, daughters of predator-exposed males prefer sons of predator-exposed fathers, this would suggest that transgenerational effects are subject to sexual selection and can affect long-term evolutionary processes by altering who reproduces. This possibility has been documented in other contexts [57–60]; for example, inheritable small RNAs in *C. elegans* (induced by high temperature) increase outcrossing and change the genetic structure of the population over time [61].

The sample size in our study was limited due to the difficulty of rearing and breeding multiple treatment groups for multiple generations, especially since sticklebacks breed annually. While our limited power largely means that we could have failed to detect more subtle patterns (Type II errors), our findings seem robust: we detected consistent behavioural patterns across multiple generations in a pool of genetically diverse males whose wild-caught ancestors were collected in different years (and likely experienced different ecological conditions). In other words, we found consistent results (within our own work, as well as with Foster *et al*. [51]) despite the opportunity for other sources of genetic and epigenetic variation to add noise, leading us to think the effects we found of F_0_ predator exposure on their sons and grandsons are robust. Another potential limitation is that we had a high level of laboratory-reared males who did not successfully hatch clutches, in both the control and predator-exposed lineages. We cannot determine whether failed clutches were due to the male or female, although visually (and anecdotally) it appeared due to poor F_1_ and F_2_ female egg quality. While we cannot eliminate the possibility that this attrition influenced our results, we did not have this attrition in the directly exposed F_0_ males and still found the same altered care patterns between control and predator-exposed males [30]. Nevertheless, future studies that replicate this design with larger sample sizes would be useful.

If it is adaptive for parents to alter parental care to reduce conspicuousness in high predation environments, then we would expect offspring who receive cues of predation risk from their parents to show a similar change in parental care behaviour if they are likely to live in a similar environment as their parents. Here, we demonstrate that paternal experience with predation risk can affect mating and paternal care behaviours for multiple generations in ways that are similar to effects induced by direct predation exposure. We assume that these effects are mediated at least initially by epigenetic changes to sperm. Given the important fitness benefits of these reproductive behaviours, these shifts in care could have important evolutionary implications for future generations by altering the quality of care received as well as potentially altering how sexual selection acts on the population. Further, this work shows that different epigenetic mechanisms do not operate in isolation of one another, but rather, cues passed via one TGP mechanism (assumed to be sperm) can induce changes in other potential TGP mechanisms (parental care). This likely has consequences for predicting when and how TGP evolves, in ways that current empirical and theoretical work have not yet examined.

## Data Availability

The data are provided in the electronic supplementary material [62].
